# The Spectral Underpinning of word2vec

**DOI:** 10.3389/fams.2020.593406

**Published:** 2020-12-03

**Authors:** Ariel Jaffe, Yuval Kluger, Ofir Lindenbaum, Jonathan Patsenker, Erez Peterfreund, Stefan Steinerberger

**Affiliations:** 1 Program in Applied Mathematics, Yale University, New Haven, CT, United States,; 2 Department of Pathology, Yale School of Medicine, New Haven, CT, United States,; 3 Interdepartmental Program in Computational Biology and Bioinformatics, New Haven, CT, United States,; 4 School of Computer Science and Engineering, The Hebrew University, Jerusalem, Israel,; 5 Department of Mathematics, University of Washington, Seattle, WA, United States

## Abstract

Word2vec introduced by Mikolov et al. is a word embedding method that is widely used in natural language processing. Despite its success and frequent use, a strong theoretical justification is still lacking. The main contribution of our paper is to propose a rigorous analysis of the highly nonlinear functional of word2vec. Our results suggest that word2vec may be primarily driven by an underlying spectral method. This insight may open the door to obtaining provable guarantees for word2vec. We support these findings by numerical simulations. One fascinating open question is whether the nonlinear properties of word2vec that are not captured by the spectral method are beneficial and, if so, by what mechanism.

## 1 INTRODUCTION

Word2vec was introduced by Mikolov et al. [1] as an unsupervised scheme for embedding words based on text corpora. We will try to introduce the idea in the simplest possible terms and refer to [1–3] for the way it is usually presented. Let {*x*_1_, *x*_2_, …, *x*_*n*_} be a set of elements for which we aim to compute a numerical representation. These may be words, documents, or nodes in a graph. Our input consists of an *n* × *n* matrix *P* with non-negative elements *P*_*ij*_, which encode, by a numerical value, the relationship between the set {*x*_*i*_}_*i*=1_ and a set of context elements {*c*_*j*_}^*n*^. The meaning of contexts is determined by the specific application, where in most cases the set of contexts is equal to the set of elements (i.e. *c*_*i*_ = *x*_*i*_ for any *i* ∈ {1, …, *n*}) [2].

The larger the value of *P*_*ij*_, the larger the connection between *x*_*i*_ and *c*_*j*_. For example, such a connection can be quantified by the probability that a word appears in the same sentence as another word. Based on *P*, Mikolov defined an energy function which depends on two sets of vector representations {*w*_1_, …, *w*_*n*_} and {*v*_1_, …, *v*_*n*_}. Maximizing the functional with respect to these sets yields $\left\{w_{1}^{*},\ldots ,w_{n}^{*}\right\}$ and $\left\{v_{1}^{*},\ldots ,v_{n}^{*}\right\}$ which can serve as a low dimensional representations for the words and contexts respectively. Ideally, this embedding should encapsulate the relations captured by the matrix *P*.

Assuming a uniform prior over the *n* elements, the energy function $L:{\mathbb{R}}^{n}\times {\mathbb{R}}^{n}\to \mathbb{R}$, introduced by Mikolov et al. [1] can be written as

(1) $$L(w,v)=\langle w,Pv\rangle -\sum_{i=1}^{n}\text{log}\left(\sum_{j=1}^{n}\text{exp}\left(w_{i}v_{j}\right)\right).$$

The exact relation between **1** and the formulation in [4] appears in Supplementary Material. Word2vec is based on maximizing this expression over all $(w,v)\in {\mathbb{R}}^{\text{n}}\times {\mathbb{R}}^{\text{n}}$

$$\left(w^{*},v^{*}\right)=\underset{\left(w,v\right)}{\text{argmax }}L(w,v).$$

There is no reason to assume that the maximum is unique. It has been observed that if *x*_*i*_ and *x*_*j*_ are similar elements in the data set (namely, words that frequently appear in the same sentence), then $v_{i}^{*}$, $v_{j}^{*}$ or $w_{i}^{*}$, $w_{j}^{*}$ tend to have similar numerical values. Thus, the values $\left\{w_{1}^{*},\ldots ,w_{n}^{*}\right\}$ are useful for embedding {*x*_1_, …, *x*_*n*_}. One could also try to maximize the symmetric loss that arises from enforcing *w* = *v* and is given by $L:{\mathbb{R}}^{\text{n}}\to \mathbb{R}$

(2) $$L(w)=\langle w,Pw\rangle -\sum_{i=1}^{n}\text{log}\left(\sum_{j=1}^{n}\text{exp}\left(w_{i}w_{j}\right)\right).$$

In Section 5 we show that the symmetric functional yields a meaningful embedding for various datasets. Here, the interpretation of the functional is straight-forward: we wish to pick $w\in {\mathbb{R}}^{n}$ in a way that makes 〈*w*, *Pw*〉 large. Assuming *P* is diagonalizable, this is achieved for *w* that is a linear combination of the leading eigenvectors. At the same time, the exponential function places a penalty over large entries in *w*.

**FIGURE 1 |:**
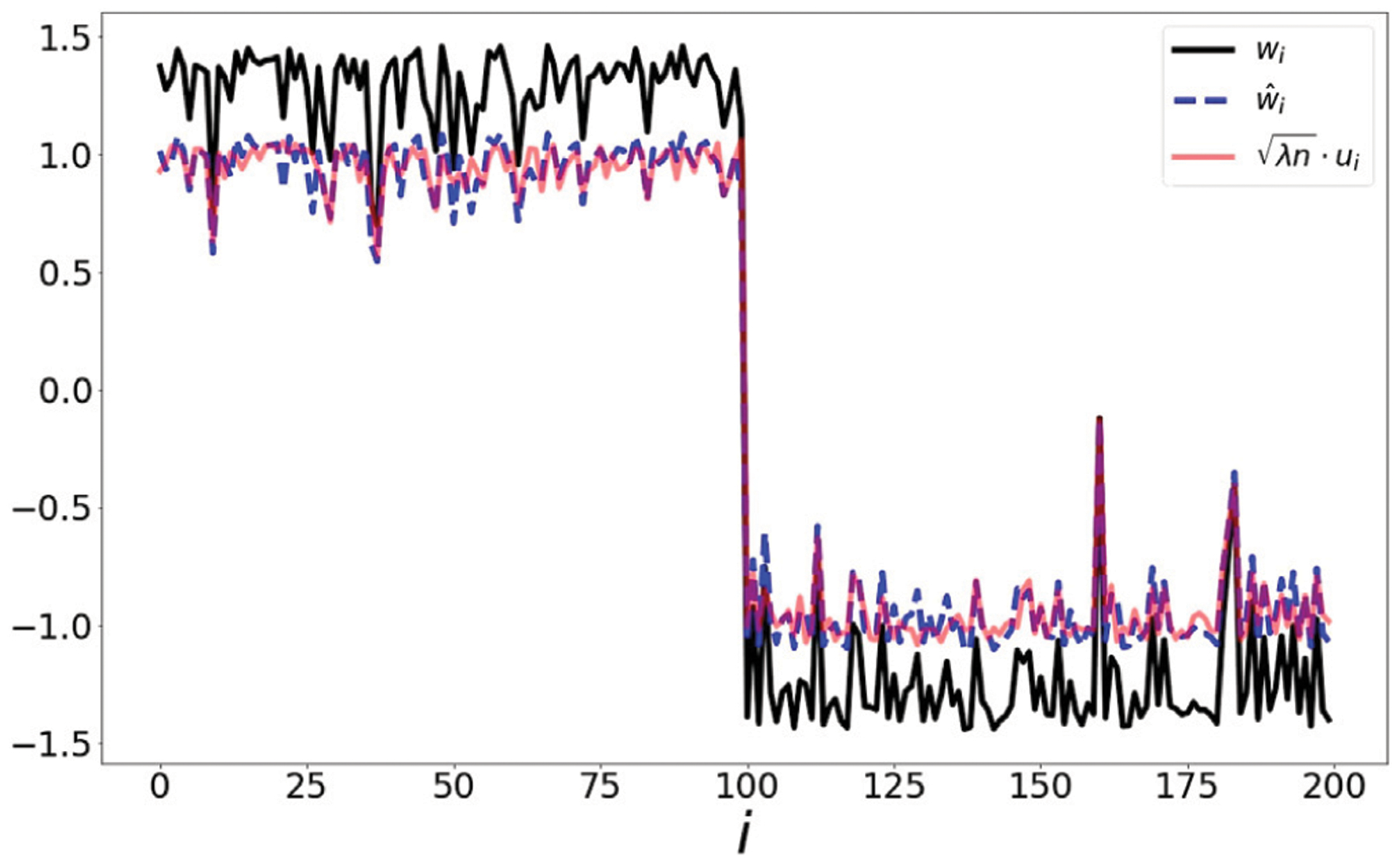
Illustration of point set drawn from two distinct Gaussian distributions. The result of maximizing over the word2vec functional (black) is closely tracked (up to scale) by the optimizer of the spectral method (blue) and the eigenvector (red). In Figure 7, we present a scatter plot comparing the values of $\hat{w}$ and $\sqrt{\lambda n}u$.

Our paper initiates a rigorous study of the energy functional *L*(*w*), however, we emphasize that a complete description of the energy landscape *L*(*w*) remains an interesting open problem. We also emphasize that our analysis has direct implications for computational aspects as well: for instance, if one were interested in maximizing the nonlinear functional, the maximum of its linear approximation (which is easy to compute) is a natural starting point. A simple example is shown in Figure 1: the underlying dataset contains 200 points in ${\mathbb{R}}^{10}$ where the first 100 points are drawn from a Gaussian distribution, and the second 100 points are drawn from a second Gaussian distribution. The matrix *P* is the row-stochastic matrix induced by a Gaussian kernel *K*_*ij*_ = exp(−∥*x*_*i*_ − *x*_*j*_∥^2^/*α*) where α is a scaling parameter discussed in Section 5. We observe that, up to scaling, the maximizer of the energy functional (black) is well approximated by the spectral methods introduced below.

## 2 MOTIVATION AND RELATED WORKS

Optimizing over energy functions such as **1** to obtain vector embeddings is done for various applications, such as words [4], documents [5] and graphs [6]. Surprisingly, very few works addressed the analytic aspects of optimizing over the word2vec functional. Hashimoto et. al. [7] derived a relation between word2vec and stochastic neighbor embedding [8]. Cotterell et al. [9] showed that when *P* is sampled according to a multinomial distribution, optimizing over **1** is equivalent to exponential family PCA [10]. If the number of elements is large, optimizing over **1** becomes impractical. As an efficient alternative, Mikolov et al. [4] suggested a variation based on negative sampling. Levy and Goldberg [11] showed that if the embedding dimension is sufficiently high, then optimizing over the negative sampling functional suggested in [4] is equivalent to factorizing the shifted Pointwise Mutual Information matrix. This work was extended in [12], where similar results were derived for additional embedding algorithms such as [3, 13, 14]. Decomposition of the PMI matrix was also justified by Arora et al. [15], based on a generative random walk model. A different approach was introduced by Landgraf [16], that related the negative sampling loss function to logistic PCA.

In this work, we focus on approximating the highly nonlinear word2vec functional by Taylor expansion. We show that in the regime of embedding vectors with small magnitude, the functional can be approximated by the spectral decomposition of the matrix *P*. This draws a natural connection between word2vec and classical, spectral embedding methods such as [17, 18]. By rephrasing word2vec as a spectral method in the “small vector limit,” one gains access to a large number of tools that allow one to rigorously establish a framework under which word2vec can enjoy provable guarantees, such as in [19, 20].

## 3 RESULTS

We now state our main results. In Section 3.1 we establish that the energy functional *L*(*w*, *v*) has a nice asymptotic expansion around $(v,w)=(0,0)\in {\mathbb{R}}^{\text{n}}\times {\mathbb{R}}^{\text{n}}$ and corresponds naturally to a spectral method in that regime. Naturally, such an asymptotic expansion is only feasible if one has some control over the size of the entries of the extremizer. We establish in Section 3.2 that the vectors maximizing the functional are not too large. The results in Section 3.2 are closely matched by numerical results: in particular, we observe that $\|w\|\sim \sqrt{n}$ in practice, a logarithmic factor smaller than our upper bound. The proofs are given in Section 4 and explicit numerical examples are shown in Section 5. In Section 6 we show empirically that the relation between word2vec and the spectral approach holds also for embedding in more than one dimension.

### 3.1 First Order Approximation for Small Data

The main idea is simple: we make an ansatz assuming that the optimal vectors are roughly of size the ∥*w*∥, ∥*v*∥ ~ 1. If we assume that the vectors *w*, *v* are fairly “typical” vectors of size ~ 1, then each entry is expected to scale approximately as ~ *n*^−1/2^. Our main observation is that this regime is governed by a regularized spectral method. Before stating our theorem, let ≲ denote the inequality up to universal multiplicative constants.

Theorem 3.1 (Spectral Expansion). If ∥*v*∥_∞_, *w*∥_∞_ ≲ *n*^−1/2^, then

$$L(w,v)=\langle w,Pv\rangle -\frac{1}{n}\left(\sum_{i=1}^{n}w_{i}\right)\left(\sum_{j=1}^{n}v_{j}\right)-\frac{1}{n}\sum_{i,j=1}^{n}\frac{w_{i}^{2}v_{j}^{2}}{2}-n\text{ log }n+O\left(n^{-1}\right).$$

Naturally, since we are interested in maximizing this quantity, the constant factor *n*log*n* plays no role. The leading terms can be rewritten as

$$\langle w,Pv\rangle -\frac{1}{n}\left(\sum_{i=1}^{n}w_{i}\right)\left(\sum_{j=1}^{n}v_{j}\right)=\langle w,\left(P-\frac{1}{n}1\right)v\rangle ,$$

where **1** is the matrix all of whose entries are 1. This suggests that the optimal *v*, *w* maximizing the quantity should simply be the singular vectors associated to the matrix $P-\frac{1}{n}1$. The full expansion has a quadratic term that serves as an additional regularizer. The symmetric case (with ansatz *v* = *w*) is particularly simple, since we have

$$L(w)=\langle w,Pw\rangle -\frac{1}{n}{\left(\sum_{i=1}^{n}w_{i}\right)}^{2}-\frac{\|w{\|}^{4}}{2n}-n\text{ log }n+O\left(n^{-1}\right).$$

Assuming *P* is similar to a symmetric matrix, the optimal *w* should be well described by the leading eigenvector of $\left(P-\frac{1}{n}1\right)$ with an additional regularization term ensuring that ∥*w*∥ is not too large. We consider this simple insight to be the main contribution of this paper, since it explains succinctly why an algorithm like word2vec has a chance to be successful. We also give a large number of numerical examples showing that in many cases the result obtained by word2vec is extremely similar to what we obtain from the associated spectral method.

### 3.2 Optimal Vectors Are Not Too Large

Another basic question is as follows: how large is the norm of the vector(s) maximizing the energy function? This is of obvious importance in practice, however, as seen in Theorem 3.1, it also has some theoretical relevance: if *w* has large entries, then clearly one cannot hope to capture the exponential nonlinearity with a polynomial expansion. Assuming ∥*P*∥ ≤ 1, the global maximizer w* of the second-order approximation

(3) $$L_{2}(w)=\langle w,Pw\rangle -\frac{1}{n}{\left(\sum_{i=1}^{n}w_{i}\right)}^{2}-\frac{\|w{\|}^{4}}{2n}-n\text{log}n,$$

satisfies

$$\|w{\|}^{*}\leq \sqrt{2n}.$$

This can be seen as follows: if ∥*P*∥ ≤ 1, then 〈*w*, *Pw*〉 ≤ ∥*w*∥^2^. Plugging in *w* = 0 shows that the maximal energy is at least size −*n*log*n*. For any vector exceeding $\sqrt{2n}$ in size, we see that the energy is less than that establishing the bound. We obtain similar boundedness properties for the fully nonlinear problem for a fairly general class of matrices.

Theorem 3.2 (Generic Boundedness.). Let $P\in {\mathbb{R}}^{\text{n}\times \text{n}}$ satisfy ∥*P*∥ < 1. Then

$$w=\text{arg}\underset{w}{\text{max}}\langle w,Pw\rangle -\sum_{i=1}^{n}\text{log}\left(\sum_{j=1}^{n}\text{exp}\left(w_{i}w_{j}\right)\right),$$

satisfies

$$\|w{\|}^{2}\leq \frac{n\text{log}n}{1-\|P\|}.$$

While we do not claim that this bound is sharp, however it does nicely illustrate that the solutions of the optimization problem must be bounded. Moreover, if they are bounded, then so are their entries; more precisely, ∥*w*∥^**2**^ ≲ *n* implies that, for ‘flat’ vectors, the typical entry is of size ≲1 and thus firmly within the approximations that can be reached by a Taylor expansion. It is clear that a condition such as ∥*P*∥ < 1 is required for boundedness of the solutions. This can be observed by considering the row-stochastic matrix

$$P=\left(\begin{array}{cc} 1-\varepsilon & \varepsilon \\ \varepsilon & 1-\varepsilon \end{array}\right).$$

Writing *w* = (*w*_1_, *w*_2_), we observe that the arising functional is quite nonlinear even in this simple case. However, it is fairly easy to understand the behavior of the gradient ascent method on the *w*_1_−axis since

$${\frac{\partial }{\partial w_{1}}L\left(w_{1},w_{2}\right)|}_{w_{2}=0}=2w_{1}\left(1-\varepsilon -\frac{e^{w_{1}^{2}}}{1+e^{w_{1}^{2}}}\right),$$

is monotonically increasing until $w_{1}\sim \pm \sqrt{\text{log}\varepsilon^{-1}}$. Therefore it is, a priori, unbounded since ε can be arbitrarily close to 0.

In practice, one often uses word2vec for matrices whose spectral norm is ∥*P*∥ = 1 and which have the additional property of being row-stochastic. We also observe empirically that the global optimizer w* has a mean value close to 0 (the expansion in Theorem 3.1 suggests why this would be the case). We achieve a similar boundedness theorem in which the only relevant operator norm is that of the operator restricted to the subspace of vectors having mean 0.

Theorem 3.3 (Boundedness for row-stochastic matrices). Let $P\in {\mathbb{R}}^{\text{n}\times \text{n}}$ be a row-stochastic matrix and let

$$P_{S}:\left\{w\in {\mathbb{R}}^{\text{n}}:w_{1}+\ldots +w_{n}=0\right\}\to {\mathbb{R}}^{\text{n}},$$

denote the restriction of P to that subspace and suppose that ∥*P*_*S*_∥ < 1. Let

$$w=\text{arg}\underset{w}{\text{max}}\langle w,Pw\rangle -\sum_{i=1}^{n}\text{log}\left(\sum_{j=1}^{n}\text{exp}\left(w_{i}w_{j}\right)\right).$$

If w has a mean value sufficiently close to 0,

$$\left|\langle w,\frac{1}{\sqrt{n}}\rangle \right|\leq \frac{1-\left\|P_{S}\right\|}{3}\|w\|,$$

where ***1*** = (1, 1, 1, …, 1), then

$$\|w{\|}^{2}\leq \frac{2n\;\text{log}n}{1-\left\|P_{S}\right\|}.$$

The 2 × 2 matrix given above, illustrates that some restrictions are necessary, in order to obtain a nicely bounded gradient ascent. There is some freedom in the choice of the constants in Theorem 3.3. Numerical experiments show that the results are not merely theoretical: extremizing vectors tend to have a mean value sufficiently close to 0 for the theorem to be applicable.

### 3.3 Outlook

Summarizing, our main arguments are as follows:

1. The energy landscape of the word2vec functional is well approximated by a spectral method (or regularized spectral method) as long as the entries of the vector are uniformly bounded. In any compact interval around 0, the behavior of the exponential function can be appropriately approximated by a Taylor expansion of sufficiently high degree.
2. There are bounds that suggests that the energy of the embedding vector scale as $\sqrt{n\text{log}n}$; this means that, for “flat” vectors, the individual entries grow at most like $\sqrt{\text{log}n}$. Presumably this is an artifact of the proof.
3. Finally, we present examples in Section 4 showing that in many cases the embedding obtained by maximizing the word2vec functional are indeed accurately predicted by the second order approximation.

This suggests various interesting lines of research: it would be nice to have refined versions of Theorems 3.2 and 3.3 (an immediate goal being the removal of the logarithmic dependence and perhaps even pointwise bounds on the entries of *w*). Numerical experiments indicate that Theorems 3.2 and 3.3 are at most a logarithmic factor away from being optimal. A second natural avenue of research proposed by our paper is to differentiate the behavior of word2vec and that of the associated spectral method: are the results of word2vec (being intrinsically nonlinear) truly different from the behavior of the spectral method (arising as its linearization)? Or, put differently, is the nonlinear aspect of word2vec that is *not* being captured by the spectral method helpful for embedding?

## 4 PROOFS

Proof of Theorem 3.1. We recall our assumption of ∥*w*∥_∞_≲*n*^−1/2^ and ∥*v*∥_∞_≲*n*^−1/2^ (where the implicit constant affects all subsequent constants). We remark that the subsequent arguments could also be carried out for any ∥*w*∥_∞_, ∥*v*∥_∞_≲*n*^*−ε*^ at the cost of different error terms; the arguments fail to be rigorous as soon as ∥*w*∥_∞_ ~ 1, since then, a priori, all terms in the Taylor expansion of *e*^*x*^ could be of roughly the same size. We start with the Taylor expansion

$$\sum_{j=1}^{n}e^{w_{i}v_{j}}=\sum_{j=1}^{n}\left(1+w_{i}v_{j}+\frac{w_{i}^{2}v_{j}^{2}}{2}+O\left(n^{-3}\right)\right)\;=n+\sum_{j=1}^{n}\left(w_{i}v_{j}+\frac{w_{i}^{2}v_{j}^{2}}{2}\right)+O\left(n^{-2}\right).$$

In particular, we note that

$$\left|\sum_{j=1}^{n}\left(w_{i}v_{j}+\frac{w_{i}^{2}v_{j}^{2}}{2}\right)\right|≲1.$$

We use the series expansion

$$\text{log}(n+x)=\text{log }n+\frac{x}{n}-\frac{x^{2}}{2n^{2}}+O{\left(\frac{\left|x\right|}{n^{3}}\right)}^{3}$$

to obtain

$$\text{log}\left(\sum_{j=1}^{n}e^{w_{i}v_{j}}\right)=\text{log }n+\frac{1}{n}\sum_{j=1}^{n}\left(w_{i}v_{j}+\frac{w_{i}^{2}v_{j}^{2}}{2}\right)-\frac{1}{2n^{2}}{\left(\sum_{j=1}^{n}w_{i}v_{j}+\frac{w_{i}^{2}v_{j}^{2}}{2}\right)}^{2}+O\left(n^{-3}\right).$$

Here, the second sum can be somewhat simplified since

$$\frac{1}{2n^{2}}{\left(\sum_{j=1}^{n}w_{i}v_{j}+\frac{w_{i}^{2}v_{j}^{2}}{2}\right)}^{2}=\frac{1}{2n^{2}}{\left(\sum_{j=1}^{n}\left(w_{i}v_{j}+O\left(n^{-2}\right)\right)\right)}^{2}\;=\frac{1}{2n^{2}}{\left(O\left(n^{-1}\right)+\sum_{j=1}^{n}w_{i}v_{j}\right)}^{2}\;=\frac{1}{2n^{2}}{\left(\sum_{j=1}^{n}w_{i}v_{j}\right)}^{2}+O\left(n^{-3}\right)\;=\frac{w_{i}^{2}}{2n^{2}}{\left(\sum_{j=1}^{n}v_{j}\right)}^{2}+O\left(n^{-3}\right)$$

Altogether, we obtain that

$$\sum_{i=1}^{n}\text{log}\left(\sum_{j=1}^{n}e^{w_{i}v_{j}}\right)=\sum_{i=1}^{n}\left(\text{log }n+\frac{1}{n}\sum_{j=1}^{n}\left(w_{i}v_{j}+\frac{w_{i}^{2}v_{j}^{2}}{2}\right)-\frac{w_{i}^{2}}{2n^{2}}{\left(\sum_{j=1}^{n}v_{j}\right)}^{2}+O\left(n^{-3}\right)\right)\;=n\text{ log }n+\frac{1}{n}\sum_{i,j=1}^{n}w_{i}v_{j}+\frac{1}{n}\sum_{i,j=1}^{n}\frac{w_{i}^{2}v_{j}^{2}}{2}-\frac{1}{n^{2}}\left(\sum_{i=1}^{n}\frac{w_{i}^{2}}{2}\right){\left(\sum_{j=1}^{n}v_{j}\right)}^{2}+O\left(n^{-2}\right).$$

Since ∥*w*∥_∞_, ∥*v*∥_∞_≲*n*^−1/2^, we have

$$\frac{1}{n^{2}}\left(\sum_{i=1}^{n}\frac{w_{i}^{2}}{2}\right){\left(\sum_{j=1}^{n}v_{j}\right)}^{2}≲n^{-1}$$

and have justified the desired expansion.

Proof of Theorem 3.2. Setting *w* = 0 results in the energy

L(w)=−nlogn.

Now, let *w* be a global maximizer. We obtain

$$-n\text{ log }n\leq \langle w,Pw\rangle -\sum_{i=1}^{n}\text{log}\left(\sum_{j=1}^{n}e^{w_{i}w_{j}}\right)$$

$$\leq \|P\|\|w{\|}^{2}-\sum_{i=1}^{n}\text{log}\left(e^{w_{i}^{2}}\right)\leq (\|P\|-1)\|w{\|}^{2}$$

which is the desired result.

Proof of Theorem 3.3. We expand the vector *w* into a multiple of the constant vector of norm 1, the vector

$$\frac{1}{\sqrt{n}}=\left(\frac{1}{\sqrt{n}},\frac{1}{\sqrt{n}},\ldots ,\frac{1}{\sqrt{n}}\right),$$

and the orthogonal complement via

$$w=\langle w,\frac{1}{\sqrt{n}}\rangle \frac{1}{\sqrt{n}}+\left(w-\langle w,\frac{1}{\sqrt{n}}\rangle \frac{1}{\sqrt{n}}\right),$$

which we abbreviate as $w=\tilde{w}+(w-\tilde{w})$. We expand,

$$\langle w,Pw\rangle =\langle \tilde{w},P\tilde{w}\rangle +\langle \tilde{w},P(w-\tilde{w})\rangle +\langle w-\tilde{w},P\tilde{w}\rangle +\langle w-\tilde{w},P(w-\tilde{w})\rangle .$$

Since *P* is row-stochastic, we have $P\tilde{w}=\tilde{w}$ and thus $\langle \tilde{w},P\tilde{w}\rangle =\|\tilde{w}{\|}^{2}$. Moreover, we have

$$\langle w-\tilde{w},P\tilde{w}\rangle =\langle w-\tilde{w},\tilde{w}\rangle =0$$

since $w-\tilde{w}$ has mean value 0. We also observe, again because $w-\tilde{w}$ has mean value 0, that

$$\langle \tilde{w},P(w-\tilde{w})\rangle =\langle \tilde{w},P_{S}(w-\tilde{w})\rangle .$$

Collecting all these estimates, we obtain

$$\frac{\langle w,Pw\rangle }{\|w{\|}^{2}}\leq \frac{\|\tilde{w}{\|}^{2}}{\|w{\|}^{2}}+\frac{\left\|\tilde{w}\right\|}{\left\|w\right\|}\frac{\left\|w-\tilde{w}\right\|}{\left\|w\right\|}\left\|P_{S}\right\|+\frac{\|w-\tilde{w}{\|}^{2}}{\|w{\|}^{2}}\left\|P_{s}\right\|.$$

We also recall the Pythagorean theorem,

$$\|\tilde{w}{\|}^{2}+\|w-\tilde{w}{\|}^{2}=\|w{\|}^{2}.$$

Abbreviating $x=\|\tilde{w}\|/w$, we can abbreviate our upper bound as

$$\frac{\langle w,Pw\rangle }{\|w{\|}^{2}}\leq x^{2}+x\sqrt{1-x^{2}}\left\|P_{S}\right\|+\left(1-x^{2}\right)\left\|P_{S}\right\|.$$

The function,

$$x\to x\sqrt{1-x^{2}}+\left(1-x^{2}\right)$$

is monotonically increasing on [0, 1/3]. Thus, assuming that

$$x=\frac{\left\|\tilde{w}\right\|}{\left\|w\right\|}\leq \frac{1-\left\|P_{S}\right\|}{3},$$

we get, after some elementary computation,

$$\frac{\langle w,Pw\rangle }{\|w{\|}^{2}}\leq {\left(\frac{1-\|P{\|}_{S}}{9}\right)}^{2}+\frac{1-\left\|P_{S}\right\|}{9}\sqrt{1-{\left(\frac{1-\left\|P_{S}\right\|}{9}\right)}^{2}}\left\|P_{S}\right\|+\left(1-{\left(\frac{1-\left\|P_{S}\right\|}{9}\right)}^{2}\right)\left\|P_{S}\right\|\leq 0.2+0.8\left\|P_{S}\right\|.$$

However, we also recall from the proof of Theorem 3.2 that

$$\sum_{i=1}^{n}-\text{log}\left(\sum_{j=1}^{n}e^{w_{i}w_{j}}\right)\leq -\|w{\|}^{2}.$$

Altogether, since the energy in the maximum has to exceed the energy in the origin, we have

$$-n\text{ log }n\leq \langle w,Pw\rangle -\sum_{i=1}^{n}\text{log}\left(\sum_{j=1}^{n}e^{w_{i}w_{j}}\right)\leq \left(0.2+0.8\left\|P_{S}\right\|\right)\|w{\|}^{2}-\|w{\|}^{2}$$

and therefore,

$$\|w{\|}^{2}\leq \frac{2n\;\text{log}n}{1-\left\|P_{S}\right\|}.$$

## 5 EXAMPLES

We validate our theoretical findings by comparing, for various datasets, the representation obtained by the following methods: i) optimizing over the symmetric functional in **1**, ii) optimizing over the spectral method suggested by Theorem 3.1 and iii) computing the leading eigenvector of $P-\frac{1}{n}1$. We denote by *w*, $\hat{w}$ and *u* be the three vectors obtained by (i)–(iii), respectively. The comparison is performed for two artificial datasets, two sets of images, a seismic dataset and a text corpus. For the artificial, image and seismic data, the matrix *P* is obtained by the following steps: we compute a pairwise kernel matrix

$$K\left(x_{i},x_{j}\right)=\text{exp}\left(-\frac{{\left\|x_{i}-x_{j}\right\|}^{2}}{\alpha }\right),$$

where α is a scale parameter set as in [21] using a max-min scale. The max-min scale is set to

(4) $$\alpha =\underset{j}{\text{max}}\left[\underset{i,i\neq j}{\text{min}}\left({\left\|x_{i}-x_{j}\right\|}^{2}\right)\right],i,j=1,\ldots n.$$

This global scale guarantees that each point is connected to at least one other point. Alternatively, adaptive scales could be used as suggested in [22]. We then compute *P* via

$$P_{ij}=K_{ij}/\sum_{l=1}^{N}K_{il}.$$

The matrix *P* can be interpreted as a random walk over the data points, (see for example [18]). Theorem 3.1 holds for any matrix *P* that is similar to a symmetric matrix, here we use the common construction from [18], but our results hold for other variations as well. To support our approximation in Theorem 3.1, Figure 7 shows a scatter plot of the scaled vector *u* vs. $\hat{w}$. In addition, we compute the correlation coefficient between *u* and $\hat{w}$ by

$$\frac{\rho (u,\hat{w})={\left(u-\mu \right)}^{T}(\hat{w}-\hat{\mu })}{\|u-\mu \|\|\hat{w}-\hat{\mu }\|,}$$

where μ and $\hat{\mu }$ are the means of *u* and $\hat{w}$ respectively. A similar measure is done for *w* and *u*. In addition, we illustrate that the norm ∥*w*∥ is comparable to $\sqrt{n}$, which supports the upper bound in Theorem 3.3.

### 5.1 Noisy Circle

**FIGURE 2 |:**
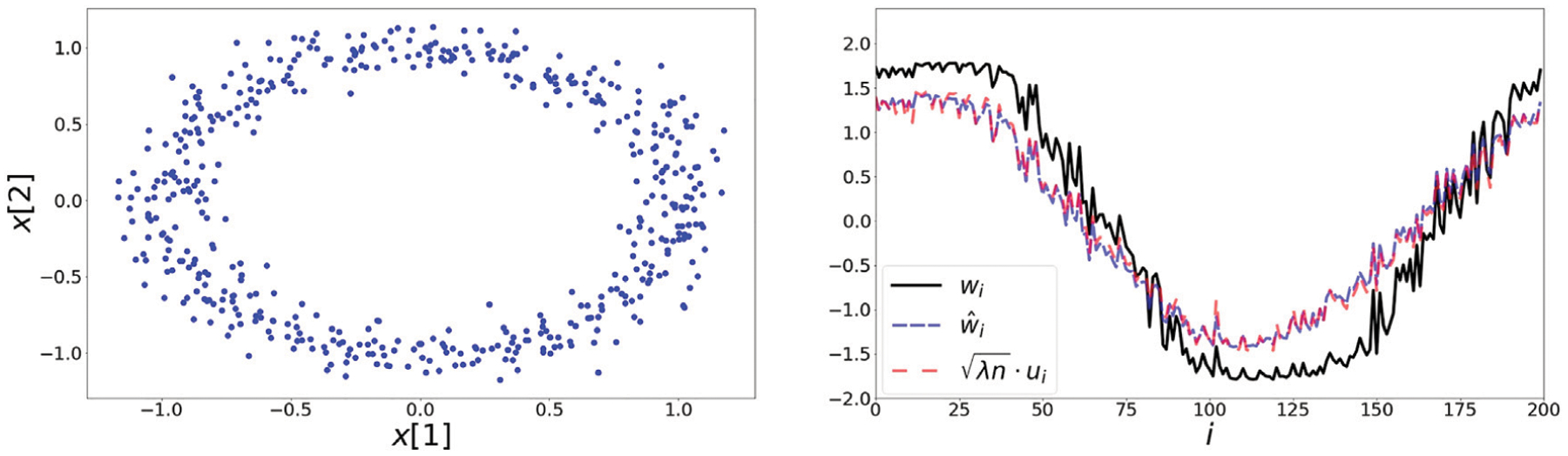
**Left:** 200 elements on the noisy circle data set. Points are generated by adding noise drawn from a two dimensional Gaussian with zero mean and a variance of 0.1. **Right:** The extracted representations based on the symmetric loss *w*, second order approximation $\hat{w}$ and leading eigenvector *u*. In Figure 7, we present a scatter plot comparing the values of $\hat{w}$ and $\sqrt{\lambda n}u$.

Here, the elements $\left\{x_{1},\ldots ,x_{200}\in {\mathbb{R}}^{2}\right\}$ are generated by adding Gaussian noise with mean 0 and *σ*^2^ = 0.1 to a unit circle (see the left panel of Figure 2). The right panel shows the extracted representations *w*, $\hat{w}$ along with the leading eigenvector *u* scaled by $\sqrt{\lambda n}$ where λ is the corresponding eigenvalue. The correlation coefficients *ρ* (*w*, *u*) and $\rho (\hat{w},u)$ are equal to 0.98, 0.99 respectively.

### 5.2 Binary MNIST

**FIGURE 3 |:**
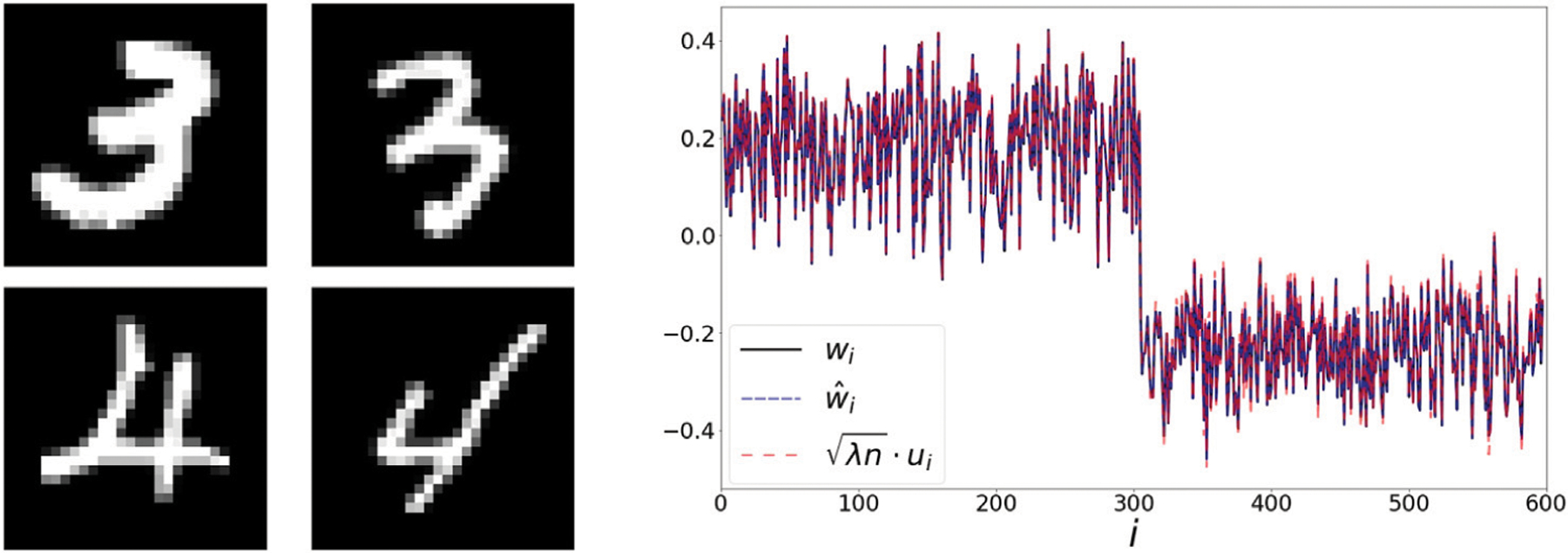
**Left:** handwritten digits from the MNIST dataset. **Right:** The extracted representations *w*, $\hat{w}$ and $\sqrt{\lambda nu}$, the leading eigenvector of $P-\frac{1}{n}1$. In Figure 7, we present a scatter plot comparing the values of $\hat{w}$ and $\sqrt{\lambda n}u$. In Figure 7, we present a scatter plot comparing the values of $\hat{w}$ and $\sqrt{\lambda n}u$.

Next, we use a set of 300 images of the digits 3 and 4 from the MNIST dataset [23]. Two examples from each category are presented in the left panel of Figure 3. Here, the extracted representations *w* and $\hat{w}$ match the values of the scaled eigenvector *u* (see right panel of Figure 3). The correlation coefficients *ρ*(*w*, *u*) and $\rho (\hat{w},u)$ are both higher than 0.999.

### 5.3 COIL100

**FIGURE 4 |:**
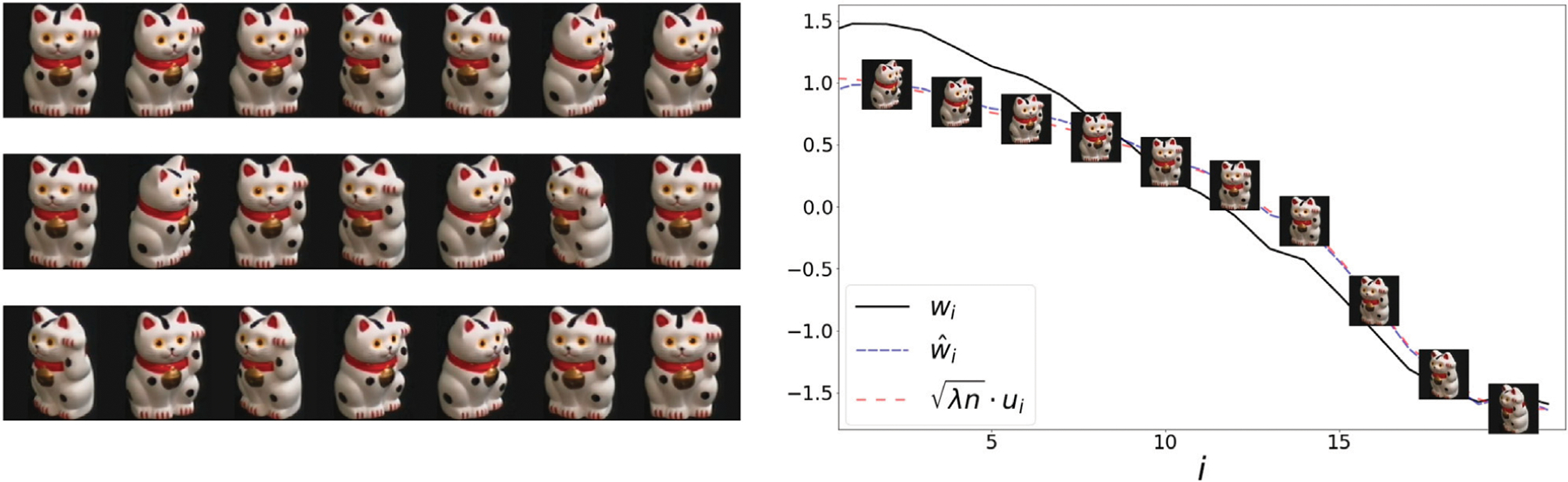
**Left:** 21 samples from COIL100 dataset. The object is captured at several unorganized angles. **Right:** The sorted values of the representations *w*, $\hat{w}$ and *u*, along with the corresponding object. Here, the representation correlates with the angle of the object. In Figure 7, we present a scatter plot comparing the values of $\hat{w}$ and $\sqrt{\lambda n}u$.

In this example, we use images from Columbia Object Image Library (COIL100) [24]. Our dataset contains 21 images of a cat captured at several pose intervals of 5 degrees (see left panel of Figure 4). We extract the embedding *w* and $\hat{w}$ and reorder them based on the true angle of the cat at every image. In the right panel, we present the values of the reordered representations *w*, $\hat{w}$ and *u* overlayed with the corresponding objects. The values of all representations are strongly correlated with the angle of the object. Moreover, the correlation coefficients *ρ* (*w*, *u*) and $\rho (\hat{w},u)$, are 0.97 and 0.99 respectively.

### 5.4 Seismic Data

**FIGURE 5 |:**
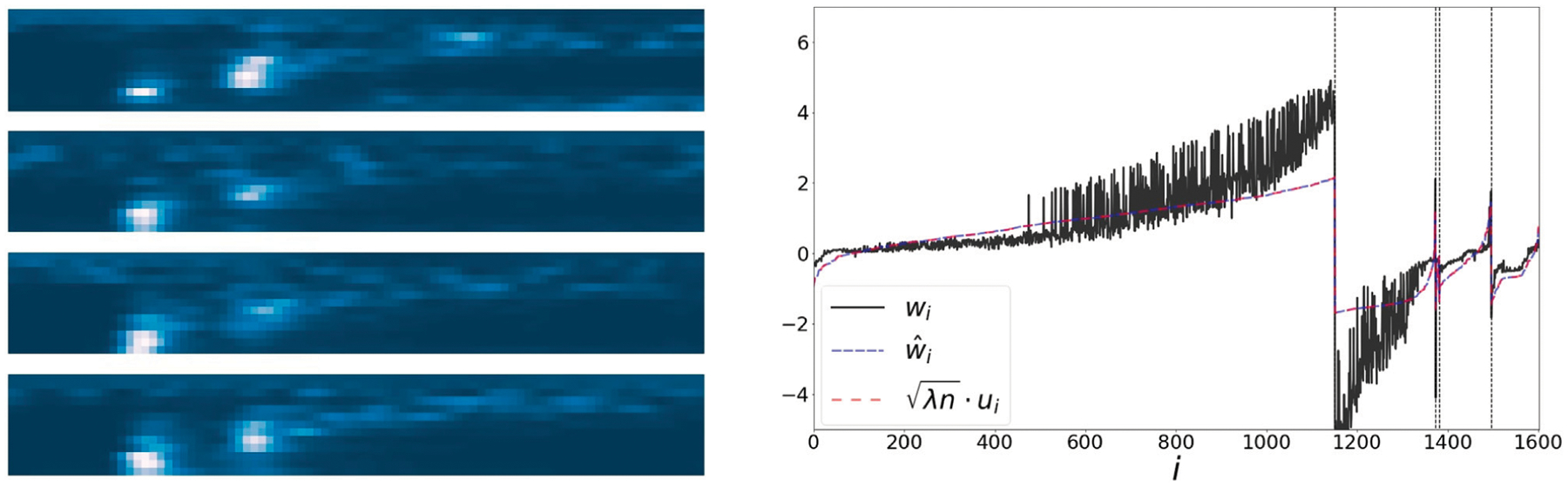
**Left:** 4 samples from the sonogram dataset, of different event types. **Right:** The values of the representations *w*, $\hat{w}$ and *u*. Dashed lines annotate the different categories of the events (based on event type and quarry location). Within each category the representations are ordered based on the value of *u*.

Seismic recordings could be useful for identifying properties of geophysical events. We use a dataset collected in Israel and Jordan, described in [25]. The data consists of 1632 seismic recordings of earthquakes and explosions from quarries. Each recording is described by a sonogram with 13 frequency bins, and 89 time bins [26] (see the left panel of Figure 5). Events could be categorized into 5 groups using manual annotations of their origin. We flatten each sonogram into a vector, and extract embeddings *w*, $\hat{w}$, and *u*. In the right panel of this figure, we show the extracted representations of all events. We use dashed lines to annotate the different categories and sort the values within each category based on *u*. The coefficient *ρ* (*w*, *v*) is equal to 0.89, and $\rho (\hat{w},v)=1$.

### 5.5 Text Data

**FIGURE 6 |:**
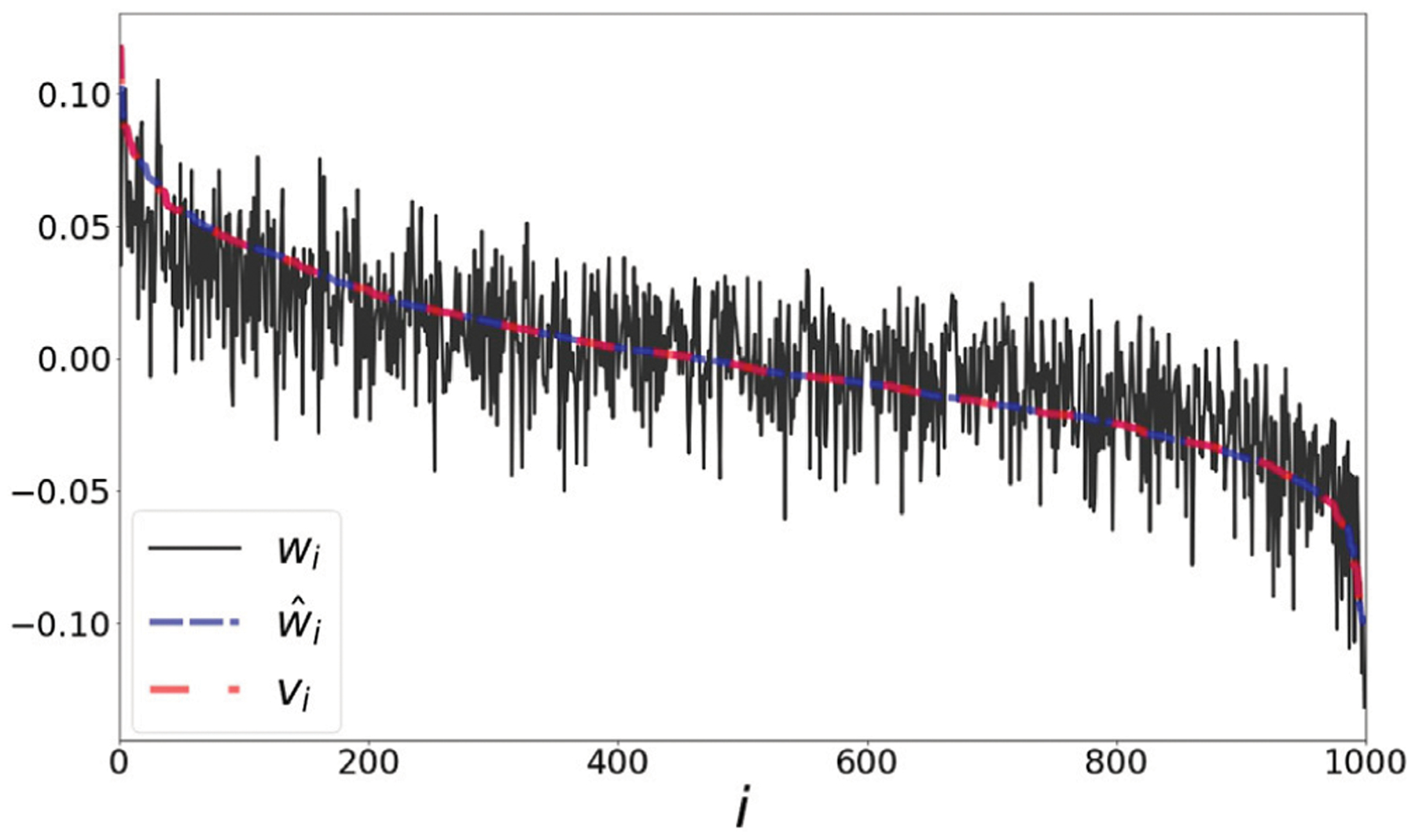
Word representation based on “Alice in Wonderland.” The values of the representations *w*, $\hat{w}$ and *v* are sorted based on the singular vector *v*. We normalized all representations to unit norm.

**FIGURE 7 |:**
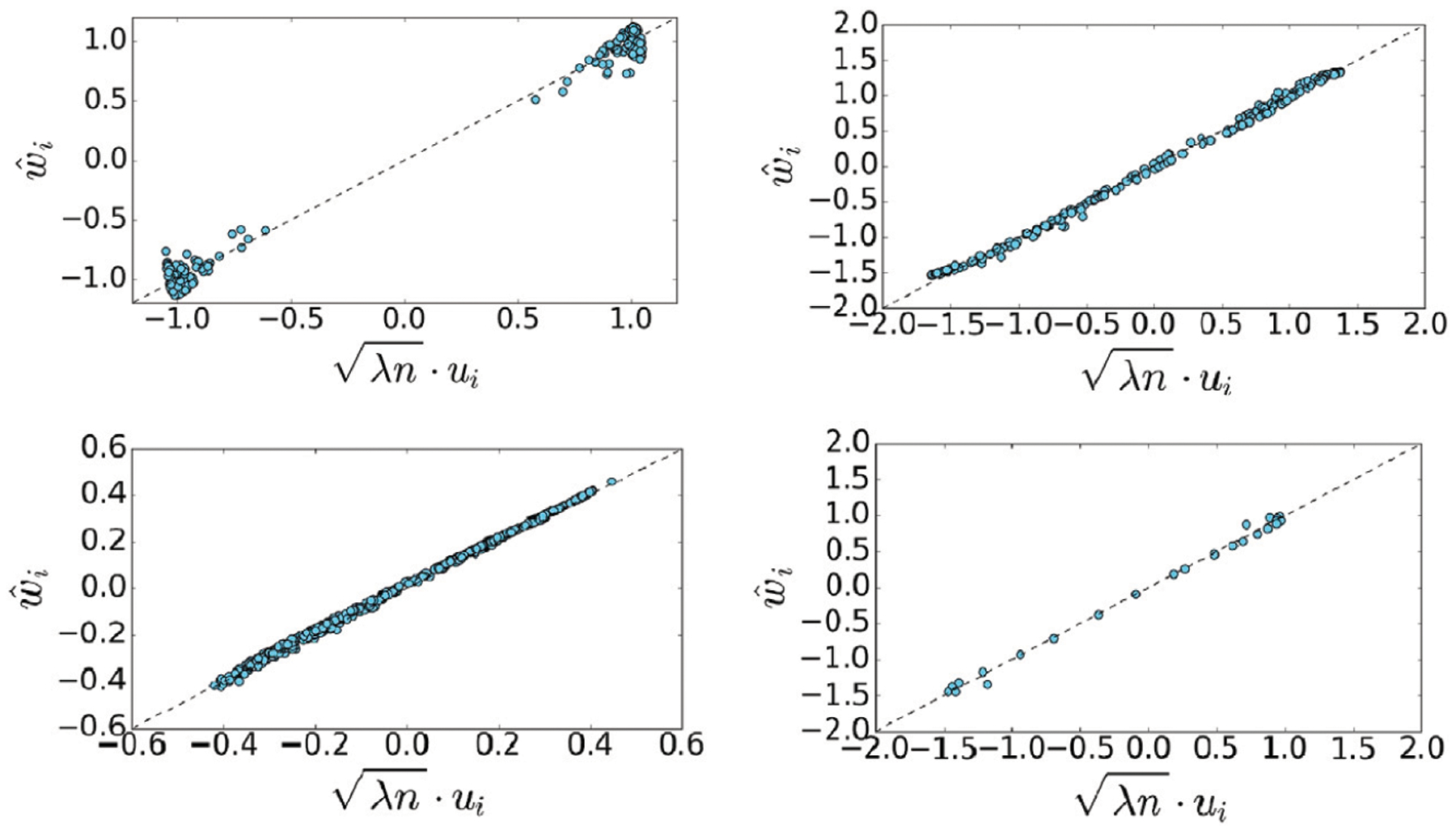
Scatter plots of the scaled eigenvector of $P-\frac{1}{n}1$, denoted by *u*, and the minimizer of the approximated functional (in Eq. 3), denoted by $\hat{w}$. From top left to bottom right, results based on data from: two Gaussian clusters, a circle, binary MNIST images and COIL100.

As a final evaluation we use a corpus of words from the book “Alice in Wonderland” as processed in [27]. To define a co-occurrence matrix, we scan the sentences using a window size covering 5 neighbors before and after each word. We subsample the top 1000 words in terms of occurrences in the book. The matrix *P* is then defined by normalizing the co-occurrence matrix. In Figure 6 we present centered and normalized versions of the representations *w*, $\hat{w}$ and the leading left singular vector *v* of $P-\frac{1}{n}1$. The coefficient *ρ* (*w*, *v*) is equal to 0.77, and $\rho (\hat{w},v)=1$.

## 6 MULTI-DIMENSIONAL EMBEDDING

In Section 3 we have demonstrated that under certain assumptions, the maximizer of the energy functional in **2** is governed by a regularized spectral method. For simplicity, we have restricted our analysis to a one dimensional symmetric representations, i.e. *w* = *v* ∈ *R*^*N*^. Here, we demonstrate empirically that this result holds even when embedding *n* elements *x*_1_, …, *x*_*n*_ in higher dimensions.

Let *w*_*i*_ ∈ *R*^*d*^ be the embedding vector associated with *x*_*i*_, where *d* ≪ *n* is the embedding dimension. The symmetric word2vec functional is given by

(5) $$L(W)=\text{Tr}\left(W^{T}PW\right)-\sum_{i=1}^{n}\text{log}\left(\sum_{j=1}^{n}\text{exp}\left(w_{i}^{T}w_{j}\right)\right),$$

where $W={\left[w_{1},\ldots ,w_{n}\right]}^{T}\in {\mathbb{R}}^{n\times d}$. A similar derivation to the one presented in Theorem 3.1 (the one dimensional case) yields the following approximation of **5**,

(6) $$L_{2}(W)=\text{Tr}\left(W^{T}\left(P-\frac{1}{n}1\right)W\right)-\frac{{\left\|W^{T}W\right\|}_{F}^{2}}{2n}-n\text{log}n.$$

Note that both the symmetric functional in **5** and its approximation in **6** are invariant to multiplying *W* with an orthogonal matrix. That is, *W* and *WR* produce the same value in both functionals, where *R* ∈ *O* (*d*).

To understand how the maximizer of **5** is governed by a spectral approach, we perform the following comparison. i) We obtain the optimizer *W* of **5** via gradient descent, and compute its left singular vectors, denoted *u*_1_, …, *u*_*d*_. ii) We compute the right singular vectors of $P-\frac{1}{n}1$, denoted by *ψ*_1_, …, *ψ*_*d*_. iii) Compute the pairwise absolute correlation values *ρ* (*u*_*i*_, *ψ*_*j*_).

We experiment on two datasets: 1) A collection of 5 Gaussians, and 2) images of hand written digits from MNIST. The transition probability matrix *P* was constructed as described in Section 5.

### 6.1 Data from Distinct Gaussians

In this experiment we generate a total of 2,500 samples, consisting of five sets of size 500. The samples in the *i*-th set are drawn independently according to a Gaussian distribution N(r⋅i⋅1,2⋅I) where **1** is a 10− dimensional all ones vector, and *r* is scalar that controls the separation between the Gaussian centers.

**FIGURE 8 |:**
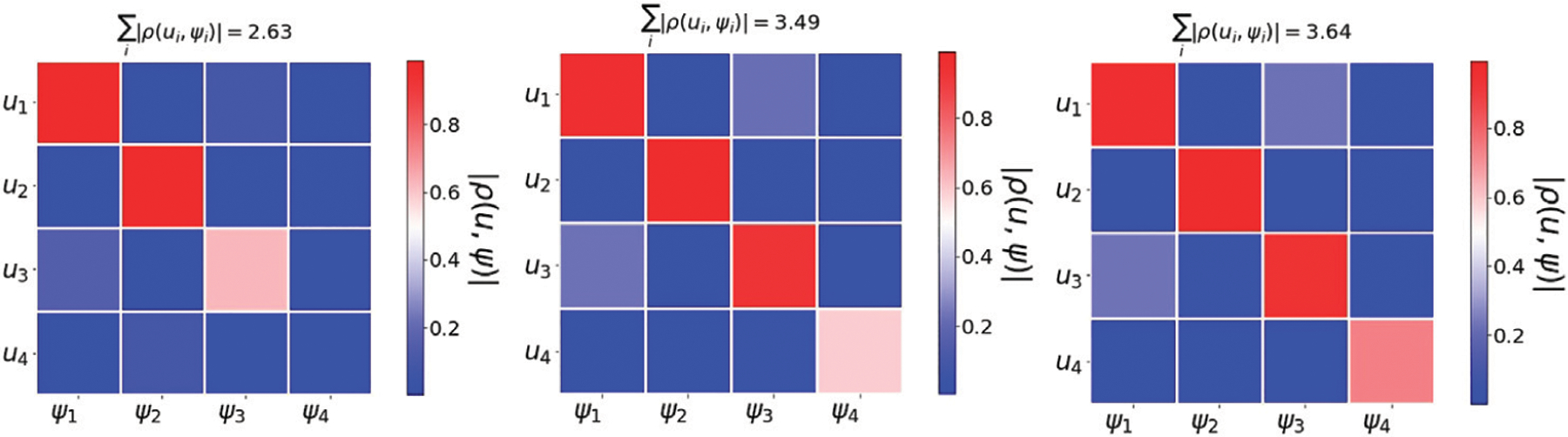
Word2vec embedding of a collection of Gaussians. We use 2,500 points generated according to five distinct Gaussians N(r⋅i⋅1,2⋅l), where **1** is a 10− dimensional all ones vector, and *r* is scalar that controls the separation between the Gaussian centers. The figure shows the absolute correlation between *u*_1_, …, *u*_4_ and *ψ*_1_, …, *ψ*_4_ for *r* = 8, 9, and 10. For each value of *r*, we compute the sum of diagonal elements in the correlation matrix. This results demonstrate the strong agreement between word2vec embedding and the spectral representation based on $P-\frac{1}{n}1$.

Figure 8 shows the absolute correlation value of the pairwise correlation between *u*_1_, …, *u*_4_ and *ψ*_1_, …, *ψ*_4_ for *r* = 8, 9, and 10. The correlation between the result obtained via the word2vec functional **5** and the right singular vectors of $P-\frac{1}{n}1$ increase when the separation between the Gaussians is high.

### 6.2 Multi-Class MNIST

The data consists of 10, 000 samples from the MNIST hand-written dataset with 1, 000 images from each digit (0 − 9). We compute a 10−dimensional word2vec embedding *W* by optimizing (5).

**FIGURE 9 |:**
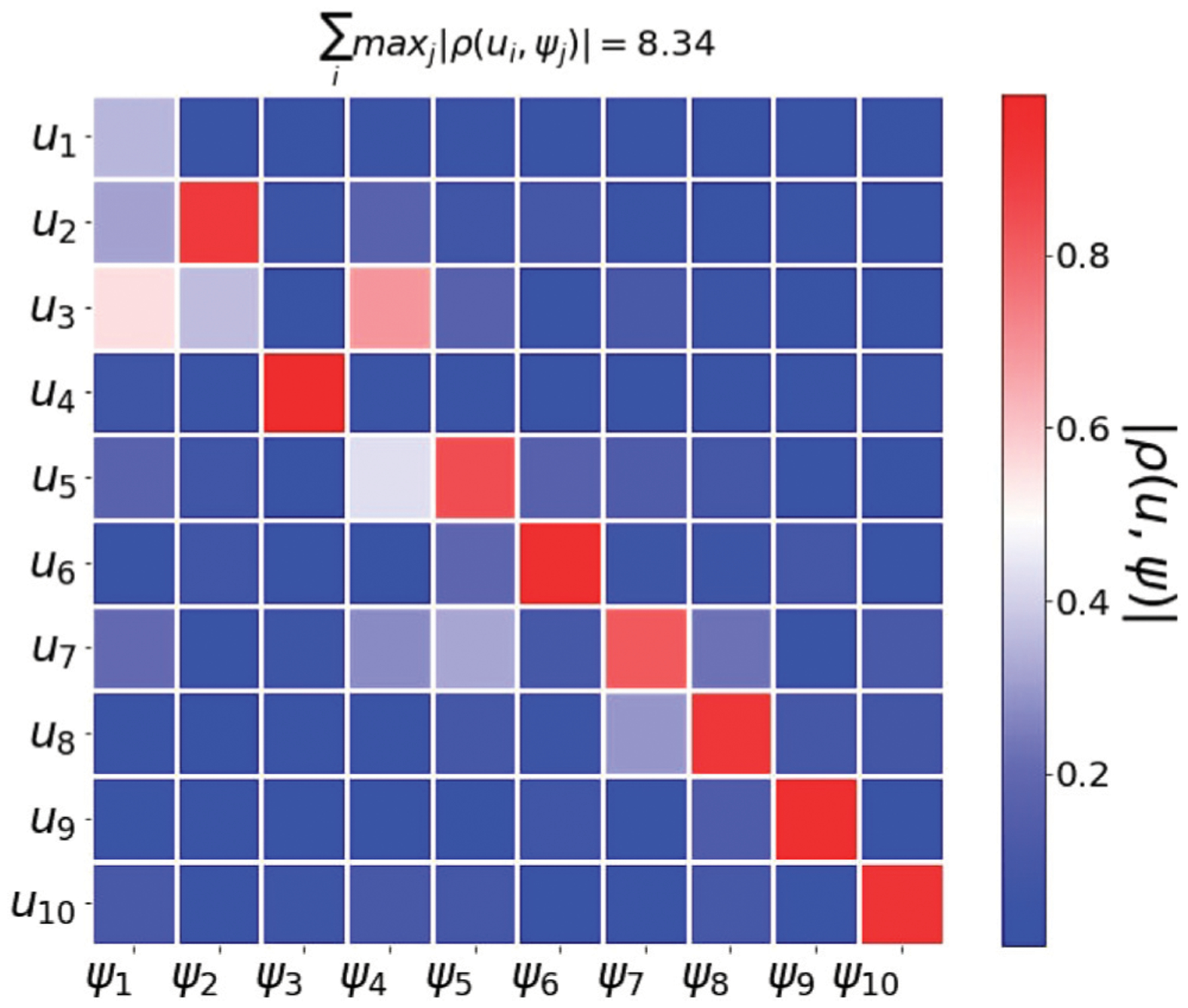
Word2vec embedding of samples from multiclass MNIST. We use 10,000 samples drawn from the MNIST handwritten dataset. The figure shows the absolute correlation between the *u*_1_, …, *u*_10_ and *ψ*_1_, …, *ψ*_10_. This results provide another empirical evidence that the eigenvectors of $P-\frac{1}{n}1$ are a good proxy for word2vec embedding.

Figure 9 shows the absolute correlation between the *u*_1_, …, *u*_10_ and *ψ*_1_, …, *ψ*_10_. As evident from the correlation matrix, the results obtained by both methods span similar subspaces.

## Supplementary Material

supp
