# Supplementary material for: The Spectral Underpinning of word2vec
Source: Front Appl Math Stat. Author manuscript; Available in PMC 2021 Sep 8. (PMC8425479; doi:10.3389/fams.2020.593406)
Supplement: supp [file NIHMS1709829-supplement-supp.pdf]

SUPPLEMENTARY MATERIAL

RELATION BETWEEN THE EXAMINED FUNCTIONAL AND THE SKIP-GRAM MODEL

The skip-gram model was introduced in Mikolov et al. (2013) as a novel method for word embedding, given a text corpora. Let $x_1, \dots, x_n$ be the vocabulary of the text, where every word x_i appears in the text at least once. We denote by $p(x_j|x_i)$ the probability that x_j appears in a window of size c around x_i. throughout the text. Let y_k be the word in location k in the text, where $k = 1, \dots, |\text{Text}|$. The goal is to set the parameters $\theta \equiv \{w_i, v_i\}_{i=1}^{n}$ as to maximize a log probability function, defined for the pattern of neighborhoods in a window of size c,

$$L(w,v) = \frac{1}{|\text{Text}|} \sum_{k=1}^{|\text{Text}|} \sum_{l \in [k-c, k+c]} \log(q(y_l|y_k;\theta)), \tag{S1}$$

where the conditional probability $q(x_i|x_j;\theta)$ is modeled using a soft-max function,

$$q(x_i|x_j;\theta) \quad = \quad \frac{\exp(\langle w_j, v_i \rangle)}{\sum_{m=1}^{n} \exp(\langle w_j, v_m \rangle)}. \tag{S2}$$

By replacing the double summation in (S1) with a double summation over the vocabulary yields

$$L(w,v) = \sum_{i=1}^{n} \sum_{j=1}^{n} p(x_i) p(x_j|x_i) \log(q(x_j|x_i;\theta)), \tag{S3}$$

where $p(x_i)$ is the prior on the word x_i. Denoting $P_{ij} = p(x_i)p(x_j|x_i)$ and inserting (S2) into (S3) yields the function in (1). If we assume a uniform probability over the vocabulary, as in the experiments in sections 5 and 6, (S3) may be simplified,

$$L(w,v) = \frac{1}{n} \sum_{i=1}^{n} \sum_{j=1}^{n} p(x_j|x_i) \log(q(x_j|x_i;\theta)).$$
